# Analysis of bacterial contamination and antibiotic residue of beef meat from city slaughterhouses in East Java Province, Indonesia

**DOI:** 10.14202/vetworld.2019.243-248

**Published:** 2019-02-12

**Authors:** Koesnoto Soepranianondo, Dhandy Koesoemo Wardhana, Budiarto, Diyantoro

**Affiliations:** 1 Department of Animal Husbandry, Faculty of Veterinary Medicine, Universitas Airlangga, Campus C Surabaya, East Java 60115, Indonesia; 2 Department of Veterinary Public Health, Faculty of Veterinary Medicine, Universitas Airlangga, Campus C Surabaya, East Java 60115, Indonesia; 3 Department of Health Science, Faculty of Vocational Studies, Universitas Airlangga, Campus B Surabaya, East Java 60286, Indonesia

**Keywords:** antibiotic residue, beef meat, city slaughterhouse, microbial contamination

## Abstract

**Aim:**

This research aimed to analyze the presence of microbial contamination and antibiotic residue in beef meat from city slaughterhouses in East Java Province, Indonesia.

**Materials and Methods:**

A total of 40 samples from city slaughterhouses were used in this study. The tests for microbial contamination used several methods including total plate count (TPC), most probable number of *Escherichia coli*, detection of *Staphylococcus aureus* using Mannitol Salt Agar media, *Salmonella* spp. detection using Bismuth Sulfite Agar media and Triple Sugar Iron Agar media, and detection of the antibiotic residue by screening tests.

**Results:**

Most of the samples were contaminated with *E. coli* (32.5% positive samples) and *S. aureus* (20.0% positive samples). The mean values of TPC and *S. aureus* contamination were lower than the maximum limit of contamination, which were 41.58 CFU/g and 13.93 CFU/g, respectively, while the mean value of *E. coli* contamination was 27.03 CFU/g which was higher than the maximum limit. A low frequency of TPC (5% positive samples) and *Salmonella* spp. contamination (2.5% positive samples) was found in meat samples. Meat samples from two of the surveyed slaughterhouses were tested positive for antibiotic residue and six of the 40 samples (15%) were also tested positive for the antibiotic residue.

**Conclusion:**

It was concluded that most of the microbial contamination in beef meat from city slaughterhouses was below the maximum limit of contamination and only two slaughterhouses were found antibiotic residues in the meat samples.

## Introduction

Meat is significant in human nutritional needs. Meat is not only rich in protein but also has complete and balanced essential amino acids [1]. The damage rate of meat depends on the number of initial microbes. The beef meat will be damaged more quickly if it has a higher number of initial microbes [2]. Meat is easily damaged because it contains approximately water 75%, protein 19%, intramuscular fat 2.5%, carbohydrate 1.2%, other substances such as vitamins, minerals, and cholesterol [3], and pH of 5.7 which is the acceptable range of contamination [4]. Microbial contamination of meat leads to spoilage, resulting in economic losses [5]. Typically, the meat of healthy animals is sterile; however, contamination may occur during the various stages of slaughter, preparation, and transportation [6]. A variety of microbes can contaminate meat, although different species may become dominant depending on factors that include pH, oxygen, water availability, and storage temperatures [6,7]. Aside from spoilage, infection of meat can be pathogenic to the consumer.

One of the critical issues in international trade is food safety [8]. Many diseases are transmitted through the food and even have led to death [9,10]. A total of 24-81 million cases of the disease have been reported each year transmitting through the food, and about 50% were related to animal and its products [11-13]. The presence of microbial contamination in food can reduce the shelf-life of food and promote foodborne illness. Foodborne pathogens originating from the animal during slaughter such as *Salmonella* spp. and *Escherichia coli* contaminate the carcass and spread to the cut or raw meat intended for further processing [14] causing a major public health problem. In line with this, 48% of all beef is in fact related to outbreaks in the United States [15].

Lack of knowledge and awareness of the public, especially for traders handling the food and distributing the meat with the requirements of safety, health, quality, and halal, can cause various diseases caused by *E. coli* and *Salmonella* spp. The slaughterhouse is the site where the meat is prepared, cut into smaller pieces, and then transported to retailers. The average Indonesian slaughterhouse may contain a variety of microbes partly due to illiteracy and lack of hygiene protocols. Therefore, the microbial analysis is essential to identify and study the level of contamination by microbes in Indonesia slaughterhouses, especially in East Java Province.

This study aimed to isolate and identify the microbial contaminants in beef meat from city slaughterhouses in East Java Province, Indonesia.

## Materials and Methods

### Ethical approval

Ethical approval for animal research was not required as live animals were not used in this study. Meat samples were purchased form government approved slaughterhouses after the animals were slaughtered.

### Meat sample collection and preparation

In this study, we selected all city slaughterhouses located in East Java Province, Indonesia. A total of 40 samples were collected from city slaughterhouses with four samples of each slaughterhouse. 100 g of raw beef meat samples were collected from each sample. The samples were obtained in the early hours of the morning and within 8 h post-slaughter to minimize the level of microbial contamination due to environmental temperatures. 10 g of collected meat samples were weighed and transferred to sterile flasks containing 90 ml of distilled water. Samples were homogenized using pestle and mortar under aseptic conditions.

### Total plate count (TPC)

About 25 g of each meat sample was weighed and transferred into Erlenmeyer containing 225 ml of 1% buffered peptone water (BPW) (Merck 1.07228.0500), homogenized for 1-2 min, and made serial dilution. Serial dilution of the meat sample was done using five sterile test tubes which were labeled 10^−1^-10^−5^ and kept in a test tube rack; 9 ml of BPW media were then measured into the five test tubes. 1 ml of diluted meat sample was introduced into the first test tube labeled 10^−1^ and mixed thoroughly, and 1 ml was taken from the first test tube and transferred to the second test tube labeled 10^−2^. This was continued until the 10^−5^ dilution was obtained. 1 ml of meat samples from 10^−3^, 10^−4^, and 10^−5^ dilutions were inoculated on each nutrient agar (Merck 1.05450.0500) and then incubated at 37°C for 18-24 h. The growing colony on the plate was counted as TPC [16].

### Most probable number (MPN) of E. coli

MPN of *E. coli* testing was done by taking 1 ml suspension-formed sample into 9 ml of BPW media with a serial dilution of three test tubes which were labeled 10^−1^-10^−3^. 1 ml of each diluted samples was transferred into five tubes containing Brilliant Green Bile Broth media (Merck 1.05454.0500) for each dilution, then inserted Durham tube, and incubated at 45.5°C for 24-48 h. Gas-produced tube was positive and suspected to be *E. coli*. The confirmation test was done by taking 1 loop of positive *E. coli* broth and inoculated on Eosin Methylene Blue Agar (EMBA) (Merck 1.01347.0500) and then incubated at 35°C for 24 h. Suspected colonies from each EMBA were transferred into Tryptone Water media (Merck 1.10859.0500) for indole testing. Calculating the amount of MPN *E. coli* was based on tubes with positive *E. coli* broth dilution using McGrady’s tables [17].

### Detection of *Staphylococcus aureus*

Detection of *S. aureus* was done by taking 0.1 ml sample from the first dilution (10^−1^) at TPC testing, then inoculated into Mannitol Salt Agar (MSA) (Merck 1.05404.0500), and incubated at 35°C for 24 h. Yellow colonies growing on MSA were described as positive *S. aureus*, while red colonies were other species of *Staphylococcus* [18].

### Detection of Salmonella spp.

Detection of *Salmonella* spp. was carried out by inserting 25 g of each sample into 225 ml of Lactose Broth and then incubated at 35°C for 24 h. 1 ml sample of the solution was inoculated into 9 ml Tetrathionate Broth (TB) (Merck 1.05285.0500) and then incubated at 35°C for 24 h. One loop of TB media was taken using inoculating loop and was streaked on Bismuth Sulfite Agar media (Merck 1.05418.0500) and then incubated at 35°C for 24 h. Typical colonies of *Salmonella* spp. were tested using Triple Sugar Iron Agar media (Merck 1.03915.0500) [19].

### Detection for antibiotic residue

Antibiotic residues in beef meat were tested using screening test by bioassay. The normal standard of inhibition zone diameter was about 20±1 mm from the diameter of the 8 mm disc paper. This is in line with Indonesian National Standard number 7424:2008 about the screening test method of antibiotic residues on meat, eggs, and milk by bioassay [20]. Making the bacterial suspension, one or two colonies of *Bacillus subtilis* were cultured in 5 ml of Nutrient Broth (Merck 1.05443.0500) at 37°C for 24 h and then homogenized using vortex until it was found to be similar to McFarland Standards. 0.2 ml bacterial suspension was inserted into a Petri dish containing Mueller-Hinton Agar (MHA) media (Merck 1.05435.0500), then spread over the surface of agar using the sterile glass spreader carefully rotating the Petri dish at an angle of 45°C at the same time, and waited 15 min to absorb the bacterial suspension [17]. The use of *B. subtilis* as a standard for the detection of antibiotic residues is mainly to detect antibiotics of the aminoglycoside group.

Samples of beef meat were sliced using a scalpel and inserted into buffer phosphate media and then centrifuged at 3000 rpm for 15 min. Holding the disc paper using forceps, the sample was pipetted on the whole disc paper and placed carefully on the surface of MHA media. Each of Petri dish was contained 5 disc papers consisting of 4 disc papers from different meat samples and 1 disc paper of antibiotic of the aminoglycoside group (Kanamycin 1.0 µg/ml). Disc paper of antibiotic was used as a positive control. Samples were incubated for 24 h at 37°C. Result calculation was based on the diameter of inhibition zone formed around the disc paper [17].

### Statistical analysis

Data from the detection of TPC, MPN *E. coli*, and *S. aureus* were analyzed by one-way analysis of variance (ANOVA) using SPSS [21] statistical software (Ver. 16.0 for Windows, SPSS Inc., Chicago, IL) continued with Duncan’s multiple range tests with 95% of significant level (p<0.05), while the data from the detection of *Salmonella* spp. and antibiotic residue were analyzed using descriptive statistics.

## Results and Discussion

Prevalence of microbial contamination and antibiotic residue is summarized in Table-1 [22]. The samples were considered positive if the TPC was higher than 1×10^6^ CFU/g based on the Indonesian National Standard. A low frequency of total bacterial count (5% positive samples) was found in meat samples. The prevalence of TPC in this study was lower than the results of the study reported by Kumar *et al*. [23]. About 29.66% of meat samples were found to exceed the maximum limit of TPC based on the Food Safety and Standards regulation, India.

**Table-1:** The prevalence of positive selected samples.

Laboratory results	Bacterial contamination				Antibiotic residue

	Total plate count*	MPN Escherichia coli**	Staphylococcus aureus***	*Salmonella* spp.	
Positive samples	2/40	13/40	8/40	1/40	6/40
% of positive samples	5	32.5	20	2.5	15

* TPC value≥1.00×10^6^ CFU/g was considered positive,

** MPN *Escherichia coli* value≥1×10^1^ CFU/g was considered positive,

*** *Staphylococcus aureus* value≥1×10^2^ CFU/g was considered positive [22]. TPC=Total plate count

On the contrary, Kim and Yim [24] reported the lower prevalence of aerobic bacteria plate count lower, in which all samples had aerobic bacteria plate count lower than 1×10^6^ CFU/g. All city slaughterhouses in this study had an average TPC lower than 1×10^6^ CFU/g with mean value of 0.26×10^6^ CFU/g. Daniyan and Unwuchiola [25] reported the higher mean value about 4.64×10^6^ CFU/g than this study, but the result of our study was higher than the results reported by Kim and Yim [24] who found that the mean values were lower than 10^2^ CFU/g. General bacterial load on meat can be measured using TPC method, which can be used in food safety monitoring. TPC results in this study were included in the good category because the mean value of TPC was below the maximum limit. A total of 5% of meat samples that had the TPC value higher than the maximum limit of microbial contamination might be caused by several factors including the knives, water, slaughterhouse floor and wall, and evisceration table [26]. Moreover, it might also be caused by poor meat handling and poor slaughterhouse environmental conditions. According to Haileselassie *et al*. [27], other factors contributed to the high bacteria load were poor standard sanitary operational procedures practiced by the slaughterhouse workers.

A total of 13 samples from 40 examined meat samples (32.5%) showed positive results for *E. coli* contamination with a number of bacteria more than 1×10^1^ CFU/g (Table-1). The mean value of *E. coli* contamination from all samples was 27.03 CFU/g which was higher than the maximum limit of *E. coli* contamination (Table-2). Based on the results of ANOVA analysis, it showed that there were significant differences in laboratory results of MPN *E. coli* among slaughterhouses. Only one slaughterhouse (S2) had significantly different on the maximum limit of *E. coli*. Higher *E. coli* prevalence rates have been reported by several researchers, which were about 40% [28], 44% [29], and 67.1% [30]. The high level of *E. coli* in beef meat might be caused by several factors including *E. coli* which is a normal flora in animal intestine so it is possible that the meat may come in contact with fecal contaminants [25,31], the nature of meat which was susceptible to *E. coli* contamination [32], high prevalence in developing countries due to large population in temporary shelter and poor hygiene [33,34], and the worker hands and the slaughtering equipment [35].

**Table-2:** The mean (±SE) number of total plate count, most probable number of *Escherichia coli*, and *Staphylococcus aureus* of city slaughterhouses.

Slaughterhouses	Total plate count (10^6^ CFU/g)	MPN of *Escherichia coli* (CFU/g)	*Staphylococcus aureus* (CFU/g)
S1	0.36^abc^±0.34	14.55^a^±5.04	21.25^ab^±16.73
S2	0.40^abc^±0.08	126.50^b^±65.66	56.25^b^±5.22
S3	0.69^cd^±0.39	14.70^a^±4.93	111.50^c^±15.71
S4	0.06^a^±0.03	68.75^ab^±57.41	0.00^a^±0.00
S5	0.01^a^±0.00	3.00^a^±0.00	0.00^a^±0.00
S6	0.19^abc^±0.14	6.10^a^±1.79	55.00^b^±18.77
S7	0.12^ab^±0.06	9.55^a^±4.72	60.00^b^±27.89
S8	0.10^ab^±0.07	4.55^a^±1.55	2.00^a^±0.71
S9	0.05^a^±0.02	9.55^a^±4.71	1.75^a^±0.75
S10	0.63^bcd^±0.12	13.00^a^±5.77	108.00^c^±7.38
Standard limit	1.00^d^±0.00	10.00^a^±0.00	100.00^c^±0.00
Mean total	0.26*	27.03*	41.58*

Different superscripts in each column differ significantly (p<0.05).

* Indicates a significant difference of p<0.05. SE=Standard error

Two slaughterhouses, labeled S3 and S10, had the mean value of *S. aureus* contamination above the maximum limit (Table-2). About 20% (8/40) of the meat samples were found *S. aureus* contamination which were higher than the maximum limit (Table-2). The prevalence number of *S. aureus* contamination in the study was slightly lower than other results reported by Schlegelova *et al*. (24.3%) [30] but was higher than reported by Bernard [36] and Kumar *et al*. [23], which were 14.81% and 8.33%, respectively. The results of *S. aureus* contamination which were higher than the maximum limit might be due to poor sanitation from slaughterhouse’s workers and slaughtering equipment. Vanderlinde *et al*. [37] isolated the positive coagulase strains of *Staphylococcus* genus from the hands of workers working in slaughterhouses. Gilbert and Harrison [38] suggested that *S. aureus* contamination might be caused by workers touching meat without using gloves or from aerosols when talking, coughing, or sneezing. Gill [39] also reported that the knives, clothing, and hands of butcher men were the sources of contamination of *S. aureus* in slaughterhouses. Contamination may come from *S. aureu*s in the nose. Further, it will contaminate the hands and contact with slaughtering equipment. For that reason, several things that need to be considered for existing slaughterhouses in East Java Province were improving hygiene and sanitation of worker and slaughtering equipment including using complete personal protective equipment, cleaning the slaughtering equipment using uncontaminated water, and maintaining personal hygiene and clothing.

Based on Table-3, the presence of *Salmonella* spp. was only observed in one beef meat sample from 40 samples tested (2.5%). Reid *et al*. [40] and da Silva *et al*. [41] obtained slightly higher results showing a prevalence of 3.3% of *Salmonella* spp. in the beef carcass and 4.53% of prevalence of *Salmonella* spp. reported by Tadesse and Gebremedhin [42]. The low prevalence of *Salmonella* spp. contamination in the beef carcass during slaughtering had already been observed in similar studies in other countries [43-45]. The lower prevalence of *Salmonella* spp. contamination found in this study might be due to the effective implementation of high hygiene and sanitation together with the Hazard Analysis and Critical Control Point (HACCP) system in the slaughterhouses. There were several factors that could explain the *Salmonella* contamination in raw beef meat at slaughterhouses, including bovine hide [46], carcass handling, and examination [42], implementation the hygienic standards of the slaughterhouses, attitude, and practice of personnel [27,47], and unsanitary environment of slaughterhouses [48].

**Table-3:** Frequency of the occurrence of *Salmonella* spp. and other bacteria on beef meat at BSA media and TSIA.

Slaughterhouses	BSA (Number of sample)		TSIA (number of sample)			Positive *Salmonella* spp. (%)
	
	No colony	With colony	*Salmonella* spp.	Other bacteria	No bacteria	
S1	0	4	1	3	0	25
S2	2	2	0	2	2	0
S3	0	4	0	4	0	0
S4	4	0	0	0	4	0
S5	3	1	0	1	3	0
S6	2	2	0	2	2	0
S7	1	3	0	3	1	0
S8	2	2	0	2	2	0
S9	4	0	0	0	4	0
S10	0	4	0	4	0	0
Frequency of occurrence	18	22	1	21	18	
Percentage of occurrence (%)			2.5	52.5	45.0	

BSA=Bismuth Sulfite Agar, TSIA=Triple Sugar Iron Agar

Results showed that the prevalence of antibiotic residue in meat samples from this study was 15% (6/40 samples examined) with the ratio of occurrence between samples containing antibiotic residue and without antibiotic residue which was 1:5.67. This finding was lower than other country antibiotic residue records in beef meat such as 22.8% in Iran [49], 30.8% in Ghana [50], 38.33% in Pakistan [51], 44% in Nigeria [52], and 57.7% in Turkey [53] but higher than the prevalence of antibiotic residue in Vietnam (7.4%) [54]. The lower prevalence of antibiotic residue in this study might be due to legislation carried by the Ministry of Agriculture Republic Indonesia about the prohibition of the use of antibiotics for growth (antibiotic growth promoter) and only for therapy with the maximum duration of 7 days and under the supervision of a veterinarian [55]. In addition, the samples that did not contain antibiotic might due to the fact that the farmers have observed and obeyed the drug withdrawal time. Therefore, when the cattle were slaughtered, their tissues did not contain any residues. This was consistent with the finding of Sanjaya [56] who suggested that antibiotic could be found in livestock products when they were harvested before the time period for drug withdrawal time exhausted in treated animals or due to antibiotics in feed. This was supported by Anggorodi [57] who stated that stopping the administration of antibiotics in a few days before animals slaughtered would eliminate the accumulation of antibiotics in the tissues. Government’s policy on animal-based food in the supervision and guidance on the safety of meat, milk, and egg products was continued to be improved. In the operational implementation, it was necessary to provide veterinary control number certificate to the animal food business, apply the good farming practice and HACCP, monitor program and surveillance for antibiotic residue, and develop the supervision for veterinary public health [57].

## Conclusion

All slaughterhouses had the mean value of TPC lower than 1×10^6^ CFU/g with the mean value of 0.26×10^6^ CFU/g. Only one slaughterhouse (S2) had the number of MPN of *E. coli* higher than normal value and significantly different. *S. aureus* contamination in two slaughterhouses (S3 and S10) had statistically significant difference higher than the maximum limit. The percentage of *Salmonella*-contaminated beef meat was about 2.5% which was only found in one slaughterhouse (S1). The ratio between the meat with residue and the meat with no residue was 1:5.67.

## Authors’ Contributions

KS, DKW, B, and D carried out the main research works, D performed the analysis of data, D and DKW prepared the manuscript, and D revised the manuscript. All of the authors have read and approved the final manuscript.
